# Ballistic strength training in adults with cerebral palsy may increase rate of force development in plantar flexors, but transition to walking remains unclear: a case series

**DOI:** 10.1186/s13102-022-00487-1

**Published:** 2022-06-03

**Authors:** Beate Eltarvåg Gjesdal, S. Mæland, B. Bogen, K. T. Cumming, V. C. Nesse, S. M. R. Torberntsson, C. B. Rygh

**Affiliations:** 1 Department of Health and Function, Western Norway University of Applied Sciences, PO Box 7030, 5020 Bergen, Norway; 2 Department of Global Public Health and Primary Care, Faculty of Medicine, University of Bergen, Bergen, Norway; 3 Faculty of Health, Welfare and Organisation, Østfold University College, Fredrikstad, Norway

**Keywords:** Gait, Muscle function, Glideboard, Explosive strength, Ultrasound, Muscle adaptation

## Abstract

**Background:**

Persons with cerebral palsy (CP) walk with reduced ankle plantar flexor power compared to typically developing. In this study, we investigated whether a ballistic strength-training programme targeting ankle plantar flexors could improve muscle strength, muscle architecture and walking function in adults with CP.

**Methods:**

Eight adults (mildly affected CP) underwent eight weeks of ballistic strength training, with two sessions per week. Before and after the intervention preferred walking speed, ankle plantar flexion rate of force development (RFD), maximal voluntary contraction (MVC), muscle thickness, pennation angle and fascicle length were measured. Data are presented for individuals, as well as for groups. Group changes were analysed using the Wilcoxon signed-rank test.

**Results:**

Data were analysed for eight participants (five women, mean age 37.9 years; six GMFCS I and two GMFCS II). Two participants increased their walking speed, but there were no significant group changes. In terms of muscle strength, there were significant group changes for RFD at 100 ms and MVC. In the case of muscle architecture, there were no group changes.

**Conclusion:**

In this study, we found that eight weeks of ballistic strength training improved ankle plantar flexor muscle strength but walking function and muscle architecture were unchanged. Larger studies will be needed to obtain conclusive evidence of the efficacy of this training method.

**Supplementary Information:**

The online version contains supplementary material available at 10.1186/s13102-022-00487-1.

## Introduction

Walking is a whole-body movement involving coordination of multiple joints and muscles [1], and the different aspects of walking can be quantified with spatiotemporal parameters, kinematics and kinetics. The various actions of the leg muscles are timed to provide support against gravity, maintain forward progression and control balance from step to step. It has been suggested that walking is influenced by intrinsic and extrinsic factors in adults with cerebral palsy (CP), which may reduce participation in daily life activities [2]. Young adults with CP find functional mobility problematic [3], and walking is often impaired due to developmental disorders that affect coordination and balance [4]. The walking function has also been found to deteriorate through adulthood in people with CP [5].

The ankle plantar flexors are essential contributors to forward propulsion, with an increased power burst in the late stance, which is decreased in persons with CP [6]. In addition, inadequate ankle push-off in this population contributes to poorer walking economy [7]. An impaired gait function in adults with CP is associated with reduced rate of force development (RFD) and increased stiffness of the ankle muscles [8]. In individuals with CP, the spastic ankle plantar flexor muscles are weaker, and they contain more fat and intramuscular connective tissue [9] than typically developing [10]. Furthermore, the reduced muscle thickness, reduced fascicle length [11] and sarcomere elongation observed in spastic CP [12] might influence RFD [13].

Muscle strength is important for walking, and even though spastic muscles respond well to strength training, it remains unclear whether walking function improves [14]. Furthermore, literature on the impact of strength training on skeletal muscle architecture in individuals with CP is sparse [15]. The American College of Sports Medicine (ACSM) has listed several factors associated with the specificity of strength training, which may be of use when designing strength-training programmes. These include the following: muscle actions involved, speed of movement, range of motion, the muscle groups to be trained, energy systems involved, intensity and training volume [16]. To improve walking in populations with neurology, a theoretical framework (based on the ACSM guidelines on the specificity and biomechanics of walking) has been proposed, targeting hip and ankle plantar flexors in strength exercises [17]. Even though power is the product of moment and angular velocity, few previous studies have explored the efficacy of velocity induced strength training and the effect on walking outcomes. Some single studies, like that of Moreau et al. [18], have found increased fascicle length in the rectus femoris muscle and improved walking speed after velocity-induced strength training. Kirk et al. [19] targeted dorsiflexors for explosive strength training that induced increased toe lift in late swing. Geertsen et al. [8] found associations between impaired gait function and reduced rapid force generation, and increased passive stiffness in dorsiflexors and plantar flexors. To our knowledge, no studies have investigated strength training with a focus on the increased speed of motion targeting the ankle plantar flexor muscles. Therefore, the aim of this study was to investigate whether eight-week ballistic strength training of the ankle plantar flexors could improve muscle strength, muscle architecture and walking function in adults with CP.

## Methods

### Participants

Participants were recruited via the Secretary General of the Norwegian CP Association, who forwarded an invitation to adult members of regional CP association. Project information was made available in local training centres and regional hospitals. Information was also posted on social media sites for users to share. Interested and potential participants were invited to an information meeting, during which the project was presented, and they were given the opportunity to ask questions.

Ten adults of Norwegian ethnicity were initially included in the study. All had completed primary school, and none had undergone surgery last year. One withdrew before the intervention started because participation was too burdensome, and one withdrew due to bursitis of the foot (considered in another study constituting the same population [20]). Six participants were classified as hemiplegic and at level I of the Gross Motor Function Classification System (GMFCS); two were classified as diplegic and at GMFCS level II (Table 1). None of the participants had previous experience with ballistic strength training. We have published another paper with the same study population, addressing the research question on whether they can perform ballistic strength training, and it will be referred to, in particular, with their experiences with the intervention [20].

**Table 1:** Participant characteristics. The most affected side in bilateral CP was based on the lowest value of maximal voluntary contraction

ID	Age (y)	Sex	GMFCS level: distribution
P1	27	W	I: right hemiplegia
P2	51	W	I: left hemiplegia
P3	28	W	I: left hemiplegia
P4	53	M	II: diplegia, left most affected
P5	34	M	II: diplegia, left most affected
P6	24	W	I: left hemiplegia
P7	30	W	I: left hemiplegia
P8	56	M	I: right hemiplegia

GMFCS: Gross Motor Function Classification System

Ethical approval was granted by the Regional Committees for Medical and Health Research Ethics (REC Western Norway; ref: 2018/2390), and all volunteers provided written informed consent before the study intervention.

### Training intervention

The participants underwent eight weeks of ballistic strength training targeting the ankle plantar flexors, twice per week in the laboratory. In week five, we introduced an additional exercise for participants to do at home, targeting the hip, the second largest power burst in walking [17]. Challenges with introducing a home exercise are discussed in a previous paper [20], and ankle plantar flexors are the primary focus in this paper. The rationale for using a ballistic training programme was to mimic ankle movement during walking, where force in the ankle muscles is generated during brief periods of time and according to the principle of specificity, with greater angle velocity. Each training session in the lab started with a general warmup of either indoor cycling or treadmill walking. Three exercises were performed with participants lying on an inclined glideboard (Total Gym RS Encompass PowerTower®): (a) jump squats, (b) single leg hopping on the paretic leg and (c) bounding on alternating legs. For a thorough description of the exercises including pictures, see “Additional file 1: Appendix 2” in Hendrey et al. [21]. Each exercise in the lab was performed for a five-minute interval, with skill acquisition as the goal. The participants were encouraged to complete as many repetitions as possible, and breaks were initiated either by the participant when needed or the researcher, if technique and/or coordination deteriorated. The sessions were monitored, and the number of repetitions and type of assistance needed were recorded, details from the sessions can be found in “Additional file 1” in Gjesdal et al. [20]. Delayed onset muscle soreness, activities besides the intervention, adverse events or other symptoms limiting participation were also recorded. The participants’ experiences of the exercises is presented more thoroughly in Gjesdal et al. [20]. All subjects were asked to continue their regular training routines.

### Strength testing

To monitor potential responses to the training, we investigated functional muscle capacity on the basis of maximal voluntary contraction (MVC) and rate of force development (RFD), walking outcomes and muscle architecture. Maximal voluntary contraction (MVC) of the plantar flexors was measured in a seated position, with the back rest reclined to approximately 30 degrees, using a stationary dynamometer (CON-TREX®MJ). Participants were instructed to perform the contraction “as quickly and forcefully as possible” and to hold for five seconds. To ensure that participants completed the task as instructed, the tester gave verbal encouragement. The participants completed three plantar flexions with maximal effort. If the participants were not able to perform with maximal effort, they were allowed an extra contraction. Data were imported offline to Microsoft Excel (version 16.50), and a torque plot was created for each trial for each subject. All torque plots were visually inspected, and those with countermovement and spikes were excluded. The trial that produced the highest MVC was used to calculate the plantar flexors’ rate of force development following onset of contraction, defined as baseline torque in the 500 ms preceding the contraction plus 3 standard deviations. Ankle plantar flexor RFD was defined as the slope of the torque-time curve (△torque/△time) in the isometric contraction at specific times (50, 100 and 200 ms), which is in accordance with previous studies [8, 19]. The force signal was smoothed by a digital fourth-order, zero-lag Butterworth filter with a 15 Hz cut-off frequency [23] in Python Launcher (v. 3.8.0).

### Walking function: gait analysis

In order to study potential changes in gait characteristics, participants underwent three-dimensional gait analysis (3DGA). They walked barefoot along a 7-m overground pathway surrounded by eight synchronous Oqus cameras (Qualisys, Gothenburg, Sweden), with a force plate (Kistler Nordic AB, Jonsered, Sweden) mounted at the halfway point. Passive reflective markers (12.5 mm) were attached to anatomical landmarks on the skin in accordance with the Plug-in-Gait marker-set. Participants were instructed to walk at their preferred walking speed. Three walking trials of both left and right foot strikes on the force-plate were processed using Qualisys Track Manager (Qualisys, Gothenburg, Sweden) and exported via the Project Automation Framework gait module (PAF gait module, Qualisys) to Visual3D (C-Motion Inc., Rockville, MD, USA). Walking speed was determined by stride length/cycle time (derived from both sides), based on a total of 12 gait cycles. Ankle joint velocity at push-off and ankle range of movement (ROM) measurements were derived from six cycles, based on only the affected/most affected side (pipeline command can be found in “Additional file 1: Appendix 2”).

### Muscle architecture

Muscle thickness, fascicle length and the pennation angle of the medial gastrocnemius were examined with 2-D mode ultrasound (GE Ultrasound Korea, Ltd., 9,, Gyeonggi-do, Korea). The participants lay in a relaxed, prone position, with a pillow at the ankle, and the resting knee and ankle joints were measured with a manual goniometer to ensure the same leg position during the pre- and post-test, as described elsewhere [11]. A water-soluble gel was applied to the ultrasound probe (5 cm MLG-15-D) to eliminate pressure on the skin. The probe was held perpendicular to the skin. The probe placement, anatomic landmarks, scars, moles and vessels were marked on acetate paper to ensure similar probe placement across baseline and post scans on the medial gastrocnemius [18]. Muscle thickness was measured with a transverse probe orientation and was defined as the distance between deep and superficial aponeuroses, as previously described [24]. Fascicle length was measured with longitudinal probe orientation, and the visible portion of the fascicle was measured within the image frame [25]. If the fascicle was not visible within the image frame, the remainder was estimated as a linear continuation of the fascicle and aponeuroses in the proximal direction [26]. Pennation angle was measured with longitudinal probe orientation and was defined as the angle formed between identified fascicles and deep aponeurosis [24]. Images were discarded if muscle contractions were detected.

### Image analyses

**Fig. 1:**
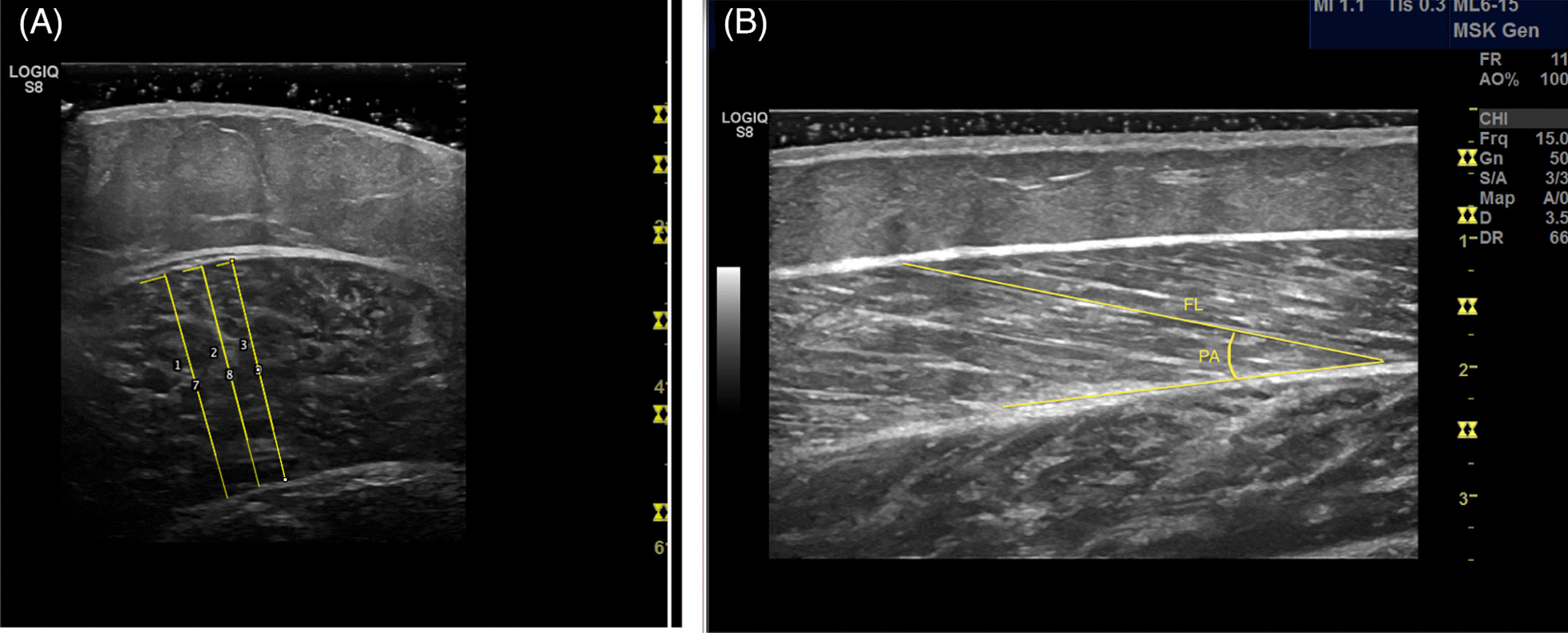
This figure demonstrates representative images of how image analysis of (**A**) muscle thickness and (**B**) fascicle length and pennation angle were performed in ImageJ

The ultrasound images were blinded for pre- and post-tests prior to analysis in ImageJ (version 1.52 k, Rasband, National Institutes of Health, USA). Two independent operators analysed the images to minimize measurement error. For each architectural parameter, two images were analysed, and the mean of the measured parameters was used for further analyses. Muscle thickness was examined as the average of two scans from fascia to fascia (Fig. 1). Fascicle length was measured from the superficial aponeurosis along the fascicle to the deep aponeurosis [25], and the remainder of the fascicle length was estimated as a linear continuation (estimated fascicle length = visible fascicle + h/sin PA), as described by Ando et al. [26]. Pennation angle was measured as the angle between the fascicle and deep aponeurosis, as demonstrated in Fig. 1. One of the assessors (VCN) did a test–retest evaluation of image measurements and found negligible measurement error.

### Analysis

Data are presented for all participants individually, with mean and standard deviation. In addition, when data are grouped, they are presented as medians and interquartile ranges. Group changes were analysed using the Wilcoxon signed-rank test, using GraphPad Prism 9.1.0 (GraphPad Software, San Diego, California, USA). P-values equal to 0.05 or lower were considered statistically significant. Data were tested for normal distribution prior to statistical analysis.

## Results

Adherence to the training sessions was 95% for participants who completed the study. Muscle strength data are missing for P3 and P6 at pre-test due to measurement error. In a prior study on the same study population, the participants were interviewed about their experiences with the intervention. Some stated that they did not feel sore or bothered by the exercises at all, while some reported that soreness and tiredness varied, and that the knee extensors in particular were sore. Muscle soreness was most pronounced in the beginning of the exercise period, suggesting that the participants adapted to the load. Delayed onset muscle soreness or other factors (like pain) did not limit the training participation. Further details of each training session is reported in Gjesdal et al. [20].

### Muscle strength measures

**Fig. 2:**
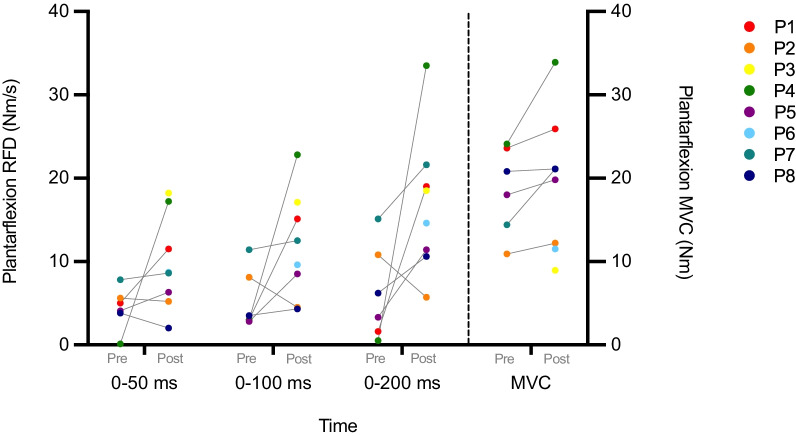
Plantar flexion RFD and MVC for participants at pre- and post-test. Pre-test values are missing for P3 and P6. Note that the left y-axis (RFD; Nm/s) refers to the results to the left side of the dashed line (RFD), while the right y-axis (MVC; Nm) refers to the results on the right side of the dashed line (MVC)

Measures of MVC revealed large variations in pre- and post-test values, both in terms of relative changes within the same subject and between individuals (Fig. 2). MVC increased in five participants, decreased in one and remained unchanged in one. Five out of six participants increased RFD at 100 and 200 ms after training. At 50 ms, only three participants showed an improving trend. Table with individual results can be found in Additional file 1: Appendix 1. There was a significant group difference for RFD at 100 ms (p = 0.03) and for MVC (p = 0.03) (Table 3).

### Walking function

Walking speed improved by more than 0.1 m/s in two participants; five were unchanged and one walked at a slower pace (Table 2). In terms of angular joint velocity, three improved, one was unchanged, and four participants were measured at lower velocity. Although walking speed is assumed to depend greatly on push-off speed in the ankle, there did not appear to be any systematic co-variation in how the participants changed with regard to angular velocity and walking speed. Ankle range of motion tended to decrease. There were no statistically significant group changes (Table 3).

**Table 2:** Walking speed (12 cycles), joint velocity at push-off and active ankle range of motion (6 cycles, only affected/most affected side) at self-selected walking speed, presented as mean and standard deviation in parentheses

	Speed (m/s)		Angular velocity at push-off (*°*/s)		Ankle RoM (*°*)	
	Pre	Post	Pre	Post	Pre	Post
P1	1.20	1.18	170 (23)	167 (20)	28 (2)	33 (3)
P2	1.12	1.38	274 (27)	275 (14)	28 (1)	25 (1)
P3	1.25	1.24	168 (13)	156 (13)	22 (1)	21 (1)
P4	0.93	0.98	242 (27)	236 (24)	27 (1)	24 (2)
P5	1.21	0.99	168 (22)	208 (24)	25 (2)	22 (2)
P6	0.97	1.42	163 (23)	177 (16)	29 (2)	25 (2)
P7	0.99	0.96	281 (9)	318 (52)	34 (1)	39 (2)
P8	0.80	0.82	167 (10)	154 (9)	24 (2)	20 (1)

m/s: metres per second; deg/s: degrees per second; ROM: range of motion

**Table 3:** Group differences in muscle architecture, walking function and muscle strength on group-level, presented as median and interquartile range (IQR)

	Pre		Post		*P *value
	Median	IQR	Median	IQR	
Muscle architecture (n = 8)					
*Muscle thickness (cm)*	1.62	1.45–1.67	1.68	1.49–1.83	0.08
*Fascicle length (cm)*	3.04	2.69–3.99	2.98	2.64–3.69	0.44
*Pennation angle (°)*	24.90	22.18–38.18	28.00	23.60–38.00	0.48
Walking function (n = 8)					
*Speed (m/s)*	1.06	0.96–1.20	1.09	0.98–1.28	0.57
*Angular velocity at push-off (°/s)*	169	167.75–250.00	192.5	164.25–245.75	0.64
*Active RoM (°)*	27.2	24.65–28.18	24.45	22.00–26.95	0.50
Muscle strength (n = 6)					
*RFD 50 (Nm/s)*	4.58	3.89–5.47	8.48	6.79–10.75	0.09
*RFD 100 (Nm/s)*	**3.29**	2.99–6.97	**10.52**	8.39–14.47	**0.03**
*RFD 200 (Nm/s)*	4.77	2.03–9.65	15.19	10.80–20.96	0.06
*MVC (Nm)*	**19.42**	15.29–22.88	**21.10**	20.11–24.69	**0.03**

Bold indicates statistically significant differences between pre- and post-test

m/s: metres per second; *°/*s: degrees per second; Nm/s: newton-metres per second; Nm: Newton meter; MVC: maximal voluntary contraction; RFD: rate of force development

### Muscle architecture

**Fig. 3:**
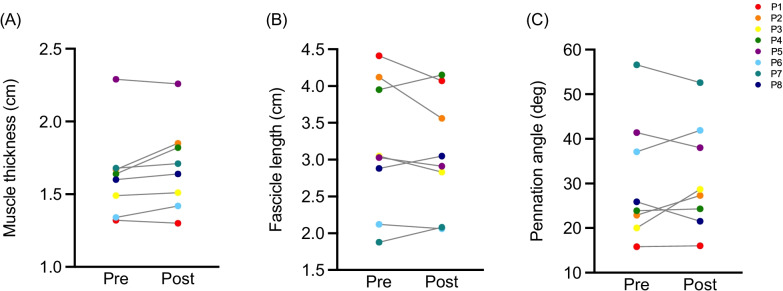
Muscle architecture at pre- and post-test in terms of (**A**) muscle thickness, (**B)** fascicle length and (**C**) pennation angle

Muscle thickness, pennation angle and fascicle length were measured to study potential changes in muscle architecture after the training intervention. Even though there were individual differences in all parameters (Fig. 3), muscle thickness was the outcome measure with the least variability. Table with individual results can be found in Additional file 1: Appendix 1. No statistically significant changes were found for any of the muscle architecture measures (Table 3).

## Discussion

The aim of this study was to investigate whether eight weeks of ballistic strength training of ankle plantar flexors could improve muscle strength, muscle architecture and walking function in adults with CP. The premise was that the training would have an impact on muscle properties, leading to improved walking (walking speed and ankle angular velocity at push-off). The main findings were that five out of six participants improved RFD and MVC after training. However, only two participants increased their walking speed after the eight weeks of intervention. Changes on group-level was found for RFD at 100 ms and MVC.

It has been asserted, in a theoretical framework, that the capacity to produce force rapidly is relevant for walking [17]. Several studies have shown that this ability is impaired in spastic CP [8, 19, 27]. We found the measured RFD post-values at the 0–200 ms time interval below other studies in adults with CP. For example, in a cross-sectional study, Geertsen et al. [8] reported RFD values that were more than twice as high as reported in this study. Their study looked at associations between impaired gait function, reduced rapid-force generation and increased passive stiffness. Also, Geertsen et al. [8] reported slightly higher MVC, which might also have affected the power produced by the plantar flexors. This may suggest that there is a threshold beyond which muscle function (mainly RFD) affects gait function in adults with CP and that the training in the present study was insufficient to reach this threshold. However, one of the participants who improved their walking speed by more than 0.1 m/s (P2) also had complete strength data, and this participant showed no remarkable changes in muscle strength. In fact, RFD at 200 ms even diminished between pre- and post-intervention. With the limited possibilities for interpretation based on a single participant, it seems that factors other than muscle strength were behind the improvement in walking speed in this participant. Furthermore, our study participants were asked to walk overground at their preferred walking speed (0.8–1.4 m/s), in contrast to Geertsen et al.’s participants [8], who walked on a treadmill at speeds of 0.1–1.3 m/s. It has previously been reported that adults with CP have a self-selected overground walking speed of approximately 0.9 m/s with bilateral CP and 1.2 m/s with unilateral CP [28]. In typically developing, angular velocity is around 300°/second at push-off at walking speeds of around 1.3-1.4 m/s [29]. Our participants’ ankle angular velocity ranged from 154–318 m/s, but five out of eight participants had angular velocities of less than 300°/second. We were unable to find an increase in walking speed similar to the increases in angular velocity that Mentiplay et al. [29] reported. One explanation is that we measured angular velocity of the ankle on the most affected side, whereas walking speed, in Mentiplay et al. (28), is derived from both ankles. Hence, angular velocity should be investigated on both sides when investigating unilateral CP (not only on the affected side) in order to fully explore the effect of an intervention.

To get an insight into potential muscle adaptations induced by training, we used ultrasound to investigate changes in muscle architecture. Fascicle length is considered to be a muscle feature with relevance to increasing muscle contraction velocity. However, we did not detect any changes in this measure. Moreau et al. [18]  reported an increase in fascicle length after 8–10 weeks of velocity-induced strength training in the rectus femoris in adults with CP, compared to conventional strength training. They concluded that spastic muscles could physiologically adapt differently to conventional and velocity-induced strength training. Moreover, the test subjects conducted the exercise tasks differently compared to our study. Participants in the mentioned study were positioned in a stationary dynamometer, which fixed limb movement, and instructors could progressively increase velocity. In contrast, our participants had to control the exercises performed on the glideboard themselves, and it was not possible to estimate the velocity at which they actually performed the exercises. A force plate mounted on the glide board’s footplate would have allowed us to give participants feedback on when they performed the exercises correctly. In a study targeting ankle plantar flexors, with rapid calf-raise training in typically developing older men, ankle plantar flexor RFD increased [30]. However, the authors could not find any changes in medial gastrocnemius muscle architecture with ultrasound, which could explain this increased RFD. They concluded that the improvements were due to neuromuscular adaptations, rather than morphological changes in muscle architecture. These responses are in accordance with our study as we measured improved RFD but no change in muscle architecture was measured with ultrasound. It should be noted that persons with CP have specific neurological damage, which makes it difficult to compare them directly with older adults. Kirk et al. [19] could not find increased ankle velocity after heavy resistance training in adults with CP. Although dorsiflexion was the main exercise in their study, the intervention also included an exercise involving a resisted calf raise. During the first few weeks of training, participants performed the exercises slowly in the eccentric phase and with full intentional acceleration in the concentric phase. The dosage was three sets with, progressively, 12–6 repetitions maximum, still, ankle-angle push-off velocity were unchanged [19].

In a recent systematic review, ballistic resistance exercises were found to be safe and feasible and to have a tendency towards efficiency in neurological populations [31]. The findings of our study are not conclusive, as we found that participants tended to increase their strength but not their walking function. We chose to conduct this as a pre-post study, and we investigated individual changes but also group changes. It is likely that our study lacked the power to detect any real changes in walking function [32]. In this study, we did not include a control group, which increases the likelihood for a placebo effect [33]. This may also be the reason why the participants reported more improvement than we were able to measure [20]. Hence, further studies should be adequately powered to obtain more solid evidence. Furthermore, we observed that participants had some difficulties in performing the exercises correctly, for example, in keeping sufficiently short contact times on the footplate of the glide board. The participants needed almost four weeks to conduct the exercises correctly, as previously reported [20]. Consequently, some of the eight-week period that was intended for strength gains were used to adapt and learn the exercises, hence frequency and duration of the intervention is discussed closer in Gjesdal et al. [20]. In athletes, there is an adaptive phase to ballistic strength training, and individuals with good baseline strength adapt more quickly than less fit individuals [34]. This may not be transferable to persons with CP, but we are of the opinion that a longer training period would have enabled participants to adapt better to the exercises. Furthermore, the rationale of this study was that a specific impairment (the inability to generate sufficient power at the right time of the gait cycle) was a limitation to walking function. In a systematic review from 2017 [14], strength training was not found to have a superior effect on, for example, walking speed. But as Williams et al. [35] contend, strengthening of, for example, the knee extensors is not task-specific to walking propulsion, but ankle plantar flexor and hip power is [17]. Therefore, we introduced home-based training targeting hip power. An extra home-based training had the potential to increase training volume, but they experienced the exercises challenging; hence only a few of them completed this training [20]. In retrospect, adding hip exercises may appear disturbing for this paper's research aim. Hence future studies are suggested to implement otherwise. Basic research is the point of departure for this article, and we assume this training did not affect the ankle plantar flexors muscle architecture or RFD.

Walking is a complex, whole-body movement, and several other factors can play a role, such as sensory and cognitive impairments. Other aspects, such as the elastic properties of tendons, may be important [36]. This was not investigated or addressed in our study. There is also evidence to suggest that walking training is better than strength training for improving walking [37]. Finally, it could be argued that habitual walking speed is an optimalization of energy cost and forward propulsion, and can even be seen as an attractor state, suggesting that it is a behaviour that is difficult to change [38, 39].

### Practical implications for adults with CP

Williams et al. [17] published a theoretical framework for improving walking function in a population with neurological disorders, discussing the general principles for strength training described in the ACSM [16] (guidelines developed for typically developing) for use when designing exercise interventions. Ballistic strength training maximizes the acceleration phase, minimizes the deceleration phase and intentionally increases the force-curve slope. Still, the basic features of muscle functioning in spastic muscles are not well documented. Instead of assuming that the same recommendations could be used in neurologically impaired persons, we recommend that a basic understanding of force production (like Henneman’s size principle and the Hill curve) in this population would be helpful in order to better understand physiological adaptations to strength training. Others have also pointed out that this is a neglected research topic, as the mechanical properties of muscle fibres are poorly understood in this population [40]. Gillett et al. have proposed strength-training interventions to investigate morphological and architectural outcomes in muscles in order to better understand the muscular response to exercise. In typically developing, RFD may be influenced by the degree of neural activation, muscle size and the muscle fibre-type composition [41]. We did not measure neural activation in our study, but others have reported that twitch interpolation of the tibialis nerve does not increase ankle plantar force output, concluding that muscle architecture is the limiting structure, and that gait rehabilitation should focus on muscle contractility [42], which supports strength training at higher velocities. With regard to muscle fibre composition, typically developing adults have an even distribution of type I and type IIa and IIx fibres [43], while persons with CP typically have more type I fibres (68–96%) [44].

## Strengths and limitations

In this study, we focused on body structures and body functioning and very little on activity and participation. Hence, important aspects of walking function were not touched upon.

The small sample size with a large age range is an important limitation of our study. With only eight participants, individual results may have had a large impact on median values. The study was also underpowered, making type 2 errors likely. With a larger sample size, the results would be easier to interpret, and they would be more generalizable. As the testing protocol was extensive (more than three hours for each participant), time and resources were a limiting factor to include more participants. In further studies, larger sample sizes should be a priority.

Ultrasound measurements depend more on operator skills than other measurements used in our study. To increase reliability, standardized criteria were followed, and two authors conducted and compared image analysis independently of one another. We did not perform any analysis of inter-tester performance, but test–retest analysis was performed for one of the assessors, and this revealed only minor measurement errors.

Choosing the force curve for analysis was difficult. The relationship between RFD and MVC is known in typically developing persons, and choosing the curve with the highest MVC is recommended [22]. However, the participants took a long time (up to five seconds) to generate MVC. The ability to produce force is impaired in individuals with CP, and further investigation of the relationship between MVC and RFD in CP is needed.

## Conclusion

Ballistic strength training targeting ankle plantar flexors may be a potential training approach to improve RFD in adults with CP, but this needs further exploration. Furthermore, assessment of whether increasing RFD might transition to walking function will require larger studies in order to reach definitive conclusions.

## Supplementary Information

**Additional file 1.** Supplementary tables (Appendix 1) and Visual3D pipeline command (Appendix 2).

## Data Availability

The datasets used and/or analysed during the current study are available from the corresponding author upon reasonable request.

## Abbreviations

CP: Cerebral palsy
GMFCS: Gross motor function classification system
MVC: Maximal voluntary contraction
RFD: Rate of force development
