# Supplementary material for: Ballistic strength training in adults with cerebral palsy may increase rate of force development in plantar flexors, but transition to walking remains unclear: a case series
Source: BMC Sports Sci Med Rehabil. 2022 Jun 3;14:101. doi: 10.1186/s13102-022-00487-1 (PMC9166478; doi:10.1186/s13102-022-00487-1)
Supplement: Supplementary file 1 — Additional file 1. Supplementary tables (Appendix 1) and Visual3D pipeline command (Appendix 2). [file 13102_2022_487_MOESM1_ESM.docx]

Appendix 1

Supplementary table 1: Muscle strength measures (MVC and RFD) of the participants affected/most affected limb at pre- and post-test.

|  | MVC (Nm) | | RFD (Nm/s) | | | | | |
| --- | --- | --- | --- | --- | --- | --- | --- | --- |
|  |  | | 0-50 ms | | 0-100 ms | | 0-200 ms | |
|  | Pre | Post | Pre | Post | Pre | Post | Pre | Post |
| P1_R | 23.6 | 25.9 | 5.0 | 11.5 | 3.0 | 15.1 | 1.6 | 19.0 |
| P2_L | 10.9 | 12.2 | 5.6 | 5.2 | 8.1 | 4.5 | 10.8 | 5.7 |
| P3_L | MD | 9.0 | MD | 18.2 | MD | 17.1 | MD | 18.5 |
| P4_R | 24.1 | 33.9 | 0.1 | 17.2 | 3.0 | 22.8 | 0.5 | 33.5 |
| P5_L | 18.0 | 19.8 | 4.1 | 6.3 | 2.8 | 8.5 | 3.3 | 11.4 |
| P6_L | MD | 11.5 | MD | MD | MD | MD | MD | MD |
| P7_L | 14.4 | 21.1 | 7.8 | 8.6 | 11.4 | 12.5 | 15.1 | 21.6 |
| P8_R | 20.8 | 21.1 | 3.8 | 2.0 | 3.5 | 4.3 | 6.2 | 10.6 |

Abbreviations: MVC: maximal voluntary contraction; RFD: rate of force development; Nm: newton-meter; Nm/s: newton-metres per second; ms: millisecond; MD: missing data

Supplementary table 2: Muscle architecture of the participants medial gastrocnemius muscle (affected/most affected limb) at pre- and post-test.

|  | Fascicle length (cm) | | Pennation angle (deg) | | Muscle thickness (cm) | |
| --- | --- | --- | --- | --- | --- | --- |
|  | Pre | Post | Pre | Post | Pre | Post |
| P1_R | 4.4 | 4.1 | 15.8 | 16.0 | 1.3 | 1.3 |
| P2_L | 4.1 | 3.6 | 22.9 | 27.3 | 1.7 | 1.9 |
| P3_L | 3.1 | 2.8 | 20.0 | 28.7 | 1.5 | 1.5 |
| P4_R | 4.0 | 4.2 | 23.9 | 24.3 | 1.6 | 1.8 |
| P5_L | 3.0 | 2.9 | 41.4 | 38.0 | 2.3 | 2.3 |
| P6_L | 2.1 | 2.1 | 37.1 | 41.9 | 1.3 | 1.4 |
| P7_L | 1.9 | 2.1 | 56.6 | 52.6 | 1.7 | 1.7 |
| P8_R | 2.9 | 3.1 | 25.9 | 21.5 | 1.6 | 1.6 |

Abbreviations: cm: centimeter; deg: degrees

Appendix 2

Ankle joint velocity

Compute_Model_Based_Data

/RESULT_NAME=A3_AnkleJointVelocity_L

/FUNCTION=JOINT_VELOCITY

/SEGMENT=LFT

/REFERENCE_SEGMENT=LSK

/RESOLUTION_COORDINATE_SYSTEM=Virtual Lab

! /USE_CARDAN_SEQUENCE=FALSE

! /NORMALIZATION=FALSE

! /NORMALIZATION_METHOD=

! /NORMALIZATION_METRIC=

! /NEGATEX=FALSE

! /NEGATEY=FALSE

! /NEGATEZ=FALSE

! /AXIS1=X

! /AXIS2=Y

! /AXIS3=Z

! /TREADMILL_DATA=FALSE

! /TREADMILL_DIRECTION=UNIT_VECTOR(0,1,0)

! /TREADMILL_SPEED=0.0

;

Compute_Model_Based_Data

/RESULT_NAME=A3_AnkleJointVelocity_R

/FUNCTION=JOINT_VELOCITY

/SEGMENT=RFT

/REFERENCE_SEGMENT=RSK

/RESOLUTION_COORDINATE_SYSTEM=Virtual Lab

! /USE_CARDAN_SEQUENCE=FALSE

! /NORMALIZATION=FALSE

! /NORMALIZATION_METHOD=

! /NORMALIZATION_METRIC=

! /NEGATEX=FALSE

! /NEGATEY=FALSE

! /NEGATEZ=FALSE

! /AXIS1=X

! /AXIS2=Y

! /AXIS3=Z

! /TREADMILL_DATA=FALSE

! /TREADMILL_DIRECTION=UNIT_VECTOR(0,1,0)

! /TREADMILL_SPEED=0.0

;

Event_Between

/NEW_EVENT_NAME=MIDSTANCE_L

! /RANGE_INSTANCE=0

/EVENT_SEQUENCE=LHS+LTO

! /EXCLUDE_EVENTS=

/FRAME_OFFSET=

! /TIME_OFFSET=

/PERCENT_OFFSET=50

;

Event_Between

/NEW_EVENT_NAME=MIDSTANCE_R

! /RANGE_INSTANCE=0

/EVENT_SEQUENCE=RHS+RTO

! /EXCLUDE_EVENTS=

/FRAME_OFFSET=

! /TIME_OFFSET=

/PERCENT_OFFSET=50

;

Metric_Minimum

/RESULT_METRIC_FOLDER=A3

/RESULT_METRIC_NAME=A3_AJV_L_Min_v2

! /APPLY_AS_SUFFIX_TO_SIGNAL_NAME=FALSE

/SIGNAL_TYPES=LINK_MODEL_BASED

! /SIGNAL_FOLDER=ORIGINAL

/SIGNAL_NAMES=A3_AnkleJointVelocity_L

/COMPONENT_SEQUENCE=ALL

/EVENT_SEQUENCE=MIDSTANCE_L+LTO+LHS

/EXCLUDE_EVENTS=

/SEQUENCE_PERCENT_START=

/SEQUENCE_PERCENT_END=

/GENERATE_MEAN_AND_STDDEV=FALSE

! /APPEND_TO_EXISTING_VALUES=FALSE

! /CREATE_GLOBAL_MINIMUM=FALSE

;

Metric_Minimum

/RESULT_METRIC_FOLDER=A3

/RESULT_METRIC_NAME=A3_AJV_R_Min_v2

! /APPLY_AS_SUFFIX_TO_SIGNAL_NAME=FALSE

/SIGNAL_TYPES=LINK_MODEL_BASED

! /SIGNAL_FOLDER=ORIGINAL

/SIGNAL_NAMES=A3_AnkleJointVelocity_R

/COMPONENT_SEQUENCE=ALL

/EVENT_SEQUENCE=MIDSTANCE_R+RTO+RHS

/EXCLUDE_EVENTS=

/SEQUENCE_PERCENT_START=

/SEQUENCE_PERCENT_END=

/GENERATE_MEAN_AND_STDDEV=FALSE

! /APPEND_TO_EXISTING_VALUES=FALSE

! /CREATE_GLOBAL_MINIMUM=FALSE

;

Ankle Range of Motion

Metric_Minimum

/RESULT_METRIC_FOLDER=A3

/RESULT_METRIC_NAME=A3_AJA_L_GaitCycle_Min

! /APPLY_AS_SUFFIX_TO_SIGNAL_NAME=FALSE

/SIGNAL_TYPES=LINK_MODEL_BASED

! /SIGNAL_FOLDER=ORIGINAL

/SIGNAL_NAMES=Left Ankle Angles

/COMPONENT_SEQUENCE=ALL

/EVENT_SEQUENCE=LHS+LTO+LHS

/EXCLUDE_EVENTS=

/SEQUENCE_PERCENT_START=

/SEQUENCE_PERCENT_END=

/GENERATE_MEAN_AND_STDDEV=FALSE

! /APPEND_TO_EXISTING_VALUES=FALSE

! /CREATE_GLOBAL_MINIMUM=FALSE

;

Metric_Maximum

/RESULT_METRIC_FOLDER=A3

/RESULT_METRIC_NAME=A3_AJA_L_GaitCycle_Max

! /APPLY_AS_SUFFIX_TO_SIGNAL_NAME=FALSE

/SIGNAL_TYPES=LINK_MODEL_BASED

! /SIGNAL_FOLDER=ORIGINAL

/SIGNAL_NAMES=Left Ankle Angles

/COMPONENT_SEQUENCE=ALL

/EVENT_SEQUENCE=LHS+LTO+LHS

/EXCLUDE_EVENTS=

/SEQUENCE_PERCENT_START=

/SEQUENCE_PERCENT_END=

/GENERATE_MEAN_AND_STDDEV=FALSE

! /APPEND_TO_EXISTING_VALUES=FALSE

! /CREATE_GLOBAL_MAXIMUM=FALSE

;

Metric_Minimum

/RESULT_METRIC_FOLDER=A3

/RESULT_METRIC_NAME=A3_AJA_R_GaitCycle_Min

! /APPLY_AS_SUFFIX_TO_SIGNAL_NAME=FALSE

/SIGNAL_TYPES=LINK_MODEL_BASED

! /SIGNAL_FOLDER=ORIGINAL

/SIGNAL_NAMES=Right Ankle Angles

/COMPONENT_SEQUENCE=ALL

/EVENT_SEQUENCE=RHS+RTO+RHS

/EXCLUDE_EVENTS=

/SEQUENCE_PERCENT_START=

/SEQUENCE_PERCENT_END=

/GENERATE_MEAN_AND_STDDEV=FALSE

! /APPEND_TO_EXISTING_VALUES=FALSE

! /CREATE_GLOBAL_MINIMUM=FALSE

;

Metric_Maximum

/RESULT_METRIC_FOLDER=A3

/RESULT_METRIC_NAME=A3_AJA_R_GaitCycle_Max

! /APPLY_AS_SUFFIX_TO_SIGNAL_NAME=FALSE

/SIGNAL_TYPES=LINK_MODEL_BASED

! /SIGNAL_FOLDER=ORIGINAL

/SIGNAL_NAMES=Right Ankle Angles

/COMPONENT_SEQUENCE=ALL

/EVENT_SEQUENCE=RHS+RTO+RHS

/EXCLUDE_EVENTS=

/SEQUENCE_PERCENT_START=

/SEQUENCE_PERCENT_END=

/GENERATE_MEAN_AND_STDDEV=FALSE

! /APPEND_TO_EXISTING_VALUES=FALSE

! /CREATE_GLOBAL_MAXIMUM=FALSE

;
